# Supplementary material for: Comprehensive Analysis of Rice Seedling Transcriptome during Dehydration and Rehydration
Source: Int J Mol Sci. 2023 May 8;24(9):8439. doi: 10.3390/ijms24098439 (PMC10179524; doi:10.3390/ijms24098439)
Supplement: Supplementary file 1 [file ijms-24-08439-s001.zip › Captions for supplementary table.pdf]

Table S1: Summary of RNA-seq libraries.

Table S2: Differentially expressed genes in group 1, 2, 3, 4, 5, and 6.

Table S3: Gene Ontology enrichment analysis of group 1, 2, 3, 4, 5, and 6.

Table S4: Gene Ontology enrichment analysis of cluster 1, 2, 3, 4, and 5.

Table S5: Kyoto Encyclopedia of Genes and Genomes pathway enrichment analysis of cluster 1, 2, 3, 4, and 5.

Table S6: Differentially expressed nitrogen metabolism-related genes in rice.

Table S7: Differentially expressed phytohormone-responsive genes in rice.

Table S8: Differentially expressed phytohormone metabolism-related genes in rice.

Table S9: Differentially expressed transcription factor-encoding genes in rice.

Table S10: Primers used in this study.
