# Supplementary material for: Comprehensive Analysis of Rice Seedling Transcriptome during Dehydration and Rehydration
Source: Int J Mol Sci. 2023 May 8;24(9):8439. doi: 10.3390/ijms24098439 (PMC10179524; doi:10.3390/ijms24098439)
Supplement: Supplementary file 1 [file ijms-24-08439-s001.zip › Supplementary Figure S1_Park and Jeong.pptx]

## Slide 1
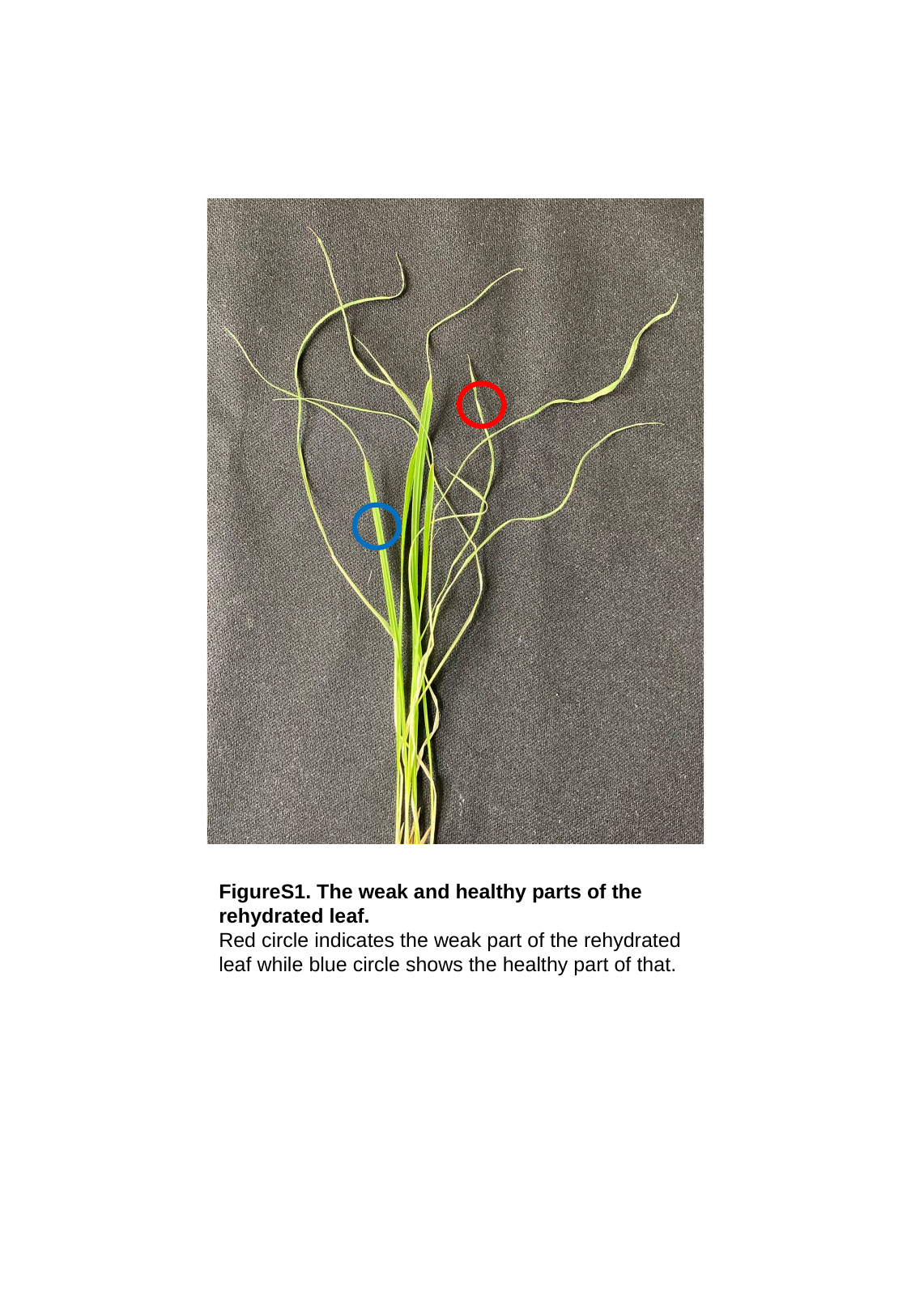

FigureS1. The weak and healthy parts of the rehydrated leaf.
Red circle indicates the weak part of the rehydrated leaf while blue circle shows the healthy part of that.
